# An Overview of Systematic Reviews of Acupuncture for Infertile Women Undergoing *in vitro* Fertilization and Embryo Transfer

**DOI:** 10.3389/fpubh.2021.651811

**Published:** 2021-04-20

**Authors:** Xia Wang, Yan Wang, Shaobin Wei, Bisong He, Yihong Cao, Nannan Zhang, Maoya Li

**Affiliations:** ^1^Hospital of Chengdu University of Traditional Chinese Medicine, Chengdu, China; ^2^Department of Clinical Medicine, Chengdu University of Traditional Chinese Medicine, Chengdu, China

**Keywords:** acupuncture, *in vitro* fertilization, systematic reviews, overview, methodological quality

## Abstract

**Background:** Currently, more and more subfertility couples are opting for combined acupuncture to improve the success rate of *in vitro* fertilization and embryo transfer (IVF-ET). However, the efficacy and safety of acupuncture in IVF-ET is still highly controversial.

**Objectives:** The purpose of this overview is to summarize evidence of essential outcomes of systematic reviews (SRs) of acupuncture in IVF-ET and evaluate their methodological quality.

**Methods:** We conducted a comprehensive literature search for relevant SRs in eight databases from inception to July 31, 2020, without language restriction. We evaluated the methodological quality of the included SRs by using A Measurement Tool to Assess Systematic Reviews 2 (AMSTAR-2), which was the latest available assessment tool. The Risk of Bias in Systematic Review (ROBIS) tool was used to assess the risk of bias in SRs. We assessed the Grades of Recommendation, Assessment, Development, and Evaluation (GRADE) score to determine the strength of evidence. We excluded the overlapping randomized controlled trials (RCTs) and performed a re-meta-analysis of the primary RCTs.

**Results:** This review included 312 original RCT studies and 65,388 participants. By using AMSTAR-2, we found that the methodological quality of 16 SRs was critically low, because they had more than one critical weakness. Our reviews showed that although the GRADE for quality of evidence profile was suboptimal, acupuncture seemed to be beneficial in increasing the pregnancy rate. Our re-meta-analysis suggested that acupuncture was superior to sham acupuncture in improving the clinical pregnancy rate (CPR) of IVF-ET with substantial heterogeneity (RR = 1.31, 95% CI: 1.13–1.52, *p* = 0.0004, *I*^2^ = 66%). No statistical difference was observed regarding the outcomes of live birth rate (LBR), ongoing pregnancy rate (OPR), biochemical pregnancy rate (BPR), and miscarriage rate (MR) between two groups. When compared with no adjunctive treatment groups, acupuncture improved CPR (RR = 1. 25, 95% CI: 1.11–1.42, *p* = 0.0003) and OPR (RR = 1. 38, 95% CI: 1.04–1.83, *p* = 0.03). Acupuncture was more superior than no adjunctive treatment in reducing MR (OR = 1.42, 95% CI: 1.03–1.95, *p* = 0.03) and BPR (RR = 1.19, 95% CI: 1.02–1.37, *p* = 0.02).

**Conclusions:** Although the evidence of acupuncture in IVF-ET is insufficient, acupuncture appears to be beneficial to increase the clinical pregnancy rate in women undergoing IVF-ET. However, there are severe heterogeneity and methodological quality defects, which limit the reliability of results. Further, high-quality primary studies are still needed.

## Introduction

Infertility is defined as failure to establish a clinical pregnancy after 12 months of regular, unprotected sexual intercourse (1, 2). The prevalence rate of infertility is 15% in the reproductive period (3). Among couples who are trying to conceive at reproductive age in China, 25% may encounter infertility (4). According to the report, female factors account for 40% of overall infertility (5). *In vitro* fertilization and embryo transfer (IVF-ET) is an effective treatment method for subfertility couples (6, 7). Worldwide, 7,079,145 cycles were carried out between 2004 and 2013, and successfully resulted in the birth of approximately 1.45 million children (8). Currently, the average success rates per IVF cycle remain low, although live birth rate gradually improved in most regions, some demonstrated declines. Fresh cycle live birth rate was highest in the USA (29%) and lowest in Japan (5%) between 2012 and 2013 (8). For many women, cycles need to be repeated to succeed. The IVF-ET cycle is time-consuming and costly. The direct and indirect costs of one cycle of IVF-ET are equivalent to $10,000 to 25,000 (9, 10), so it is essential to enhance the success rate of IVF-ET. Therefore, many patients try to seek alternative therapies to improve IVF-ET outcomes, including acupuncture. Acupuncture involves the insertion of metallic needles into the body. The application of acupuncture through stimulating certain acupoints on the human body is to activate the meridians and to regulate the function of qi and blood so as to prevent and treat disease. In the USA, 47% of infertile women engaged in acupuncture during IVF-ET treatment (11). The mechanisms of acupuncture in IVF-ET have been reported to enhance hormonal balance (12), inhibit uterine motility (13), increase blood flow to uterine and ovarian areas (14), and downregulate various stress responses (15–17).

Nowadays, some systematic reviews (SRs) of acupuncture in IVF-ET have been published to evaluate its effectiveness and safety. However, no consensus has been reached. Some reviews indicated that acupuncture might provide benefits in improving reproductive outcomes (18–29). Others found no statistically significant difference in clinical pregnancy or live birth when compared with a control (30–33). The quality of evidence is unclear. Therefore, our study aims to assess the methodological and reporting quality of SRs concerning the effectiveness of acupuncture for infertile women undergoing IVF-ET, and to provide beneficial recommendations for the implementation of SRs.

## Method

### Protocol and Registration

The protocol of this study was registered in PROSPERO with the registration number CRD42020201238 (https://www.crd.york.ac.uk/PROSPERO/).

### Search Strategy for Identification of Studies

Electronic literature was searched in the following databases from inception to July 31, 2020, without language restrictions: including four international electronic databases (Pubmed, EMBASE, Cochrane Library, and Web of Science) and four Chinese electronic databases (CNKI, Sino, Wan Fang, and VIP database). Unpublished conference proceedings relevant to infertile women undergoing IVF-ET were reviewed, if available. MeSH items or free words included: (*In vitro* fertilization OR Intracytoplasmic sperm injection OR Embryo transfer OR Assisted reproductive techniques) AND (systematic review OR Meta-analysis) AND (Acupuncture OR Acupuncture therapy OR Acupuncture points OR Electroacupuncture OR Acupuncture Analgesia). The search strategy of EMBASE is shown in Appendix and modified to suit other databases.

### Inclusion and Exclusion Criteria

#### Study Participants

Female patients diagnosed with infertility and undergoing IVF-ET would be included. There were no restrictions in diagnosis criteria or participant age. IVF-ET with intracytoplasmic sperm injection (ICSI) was allowed.

#### Study Intervention

Treatment with acupuncture must be used as the primary intervention measure. All types of acupuncture (i.e., traditional acupuncture, electro-acupuncture, auricular acupuncture, laser acupuncture) were included. It could be treated with acupuncture alone, or combined with medicine, or with other treatments, regardless of the frequency or duration of the treatment.

#### Study Comparison

The control interventions included placebo (sham) acupuncture or no adjunctive treatment, or western medicine treatment, or rehabilitation exercise, or Chinese herb medicine, or Tui-na massage, or other convenient controls. Systematic reviews comparing different types of needle were excluded.

#### Study Outcome Measures

The primary outcomes were clinical pregnancy rate (CPR), live birth rate (LBR), biochemical pregnancy rate (BPR), ongoing pregnancy rate (OPR), and miscarriage rate (MR). The secondary outcomes were adverse events.

#### Study Design

Systematic reviews containing more than one randomized controlled trial (RCT) were included. Non-RCT SRs, review comments, overviews of SRs, editorials, and guidelines were excluded.

### Selection of Studies and Data Extraction

According to the intended inclusion and exclusion criteria, the articles were screened independently by two reviewers (XW and YW). Another reviewer (SBW) was involved and made a decision when two authors showed a difference of opinion. One reviewer (XW) used a standardized form to extract the following information from the included articles; another author (YW) reviewed the extracted document. Data extraction included researcher, publication time, number of RCTs enrolled, data synthesis methods, quality assessment tool for RCTs, adverse effects, characteristics of interventions and control groups, primary and secondary outcomes, evaluation criteria of methodology, main results, and conclusions.

### Assessment of Systematic Reviews

Two independent reviewers (XW and YW) evaluated the quality of the included SRs. Before the evaluation, each topic of the assessment tools was intensively discussed to reach a consensus.

#### Assessment of Methodological Quality

The methodological quality of the included reviews was assessed by using A Measurement Tool to Assess Systematic Reviews 2 (AMSTAR-2), which was the latest available assessment tool. The AMSTAR-2 contains 16 domains, of which domains 2, 4, 7, 9, 11, 13, and 15 were the critical items, and the items should not be used to derive an overall score (34). We adopted the rating process based on the identification of critical domains. AMSTAR-2 domains are shown in Supplementary Figure 2.

#### Assessment of Bias

Risk of Bias in Systematic Review (ROBIS) is a new tool for assessing the risk of bias in systematic reviews (35). The four critical elements were “study eligibility criteria,” “identification and selection of studies,” “data collection and study appraisal” and “synthesis and findings.” Each domain was evaluated as “high risk,” “low risk,” or “unclear risk.”

#### The Quality of Evidence

For each significant result, we gave a score of 4 because these were based on randomized trials and assessed limitations that might have reduced the evidence's quality (36). We deducted points if there were: study limitations; sparse data on an outcome of interest, inconsistent results, indirectness of evidence, or imprecision. According to the Grades of Recommendation, Assessment, Development, and Evaluation (GRADE) score, the quality of evidence was classified into high, moderate, low, and very low (36, 37).

### Strategy for Data Synthesis

To assess the combined effects of the included reviews, we performed a re-meta-analysis of the main outcomes. The effects of acupuncture on CPR, LBR, BPR, OPR, and MR were observed in women undergoing IVF-ET. Considering the overlap of some primary RCTs, the two reviewers listed the trials for each SR and then excluded the overlapping trials. All data were dichotomous. When no significant heterogeneity could be observed (*I*^2^ < 50%), a fixed-effects model was applied and results were pooled. If there was significant heterogeneity (*I*^2^ ≥ 50%), the random-effects model was applied correctly, and expressed as relative risk (RR). The Forest plots were performed with RevMan5.3 software.

We also performed a subgroup analysis of several factors that might influence the CPR of acupuncture. This included: (1) age (≥35 or <35 years); (2) duration of infertility (≥4 or <4 years); (3) number of embryos transferred (≥2 or <2); (4) type of sham (invasive vs. non-invasive); and (5) sham points (yes or no). The information of the variables for subgroup analysis are shown in Supplementary Table 1. Sensitivity analysis was performed to explore whether the overall conclusions were affected.

## Results

### Results on Literature Search and Selection

**Figure 1:**
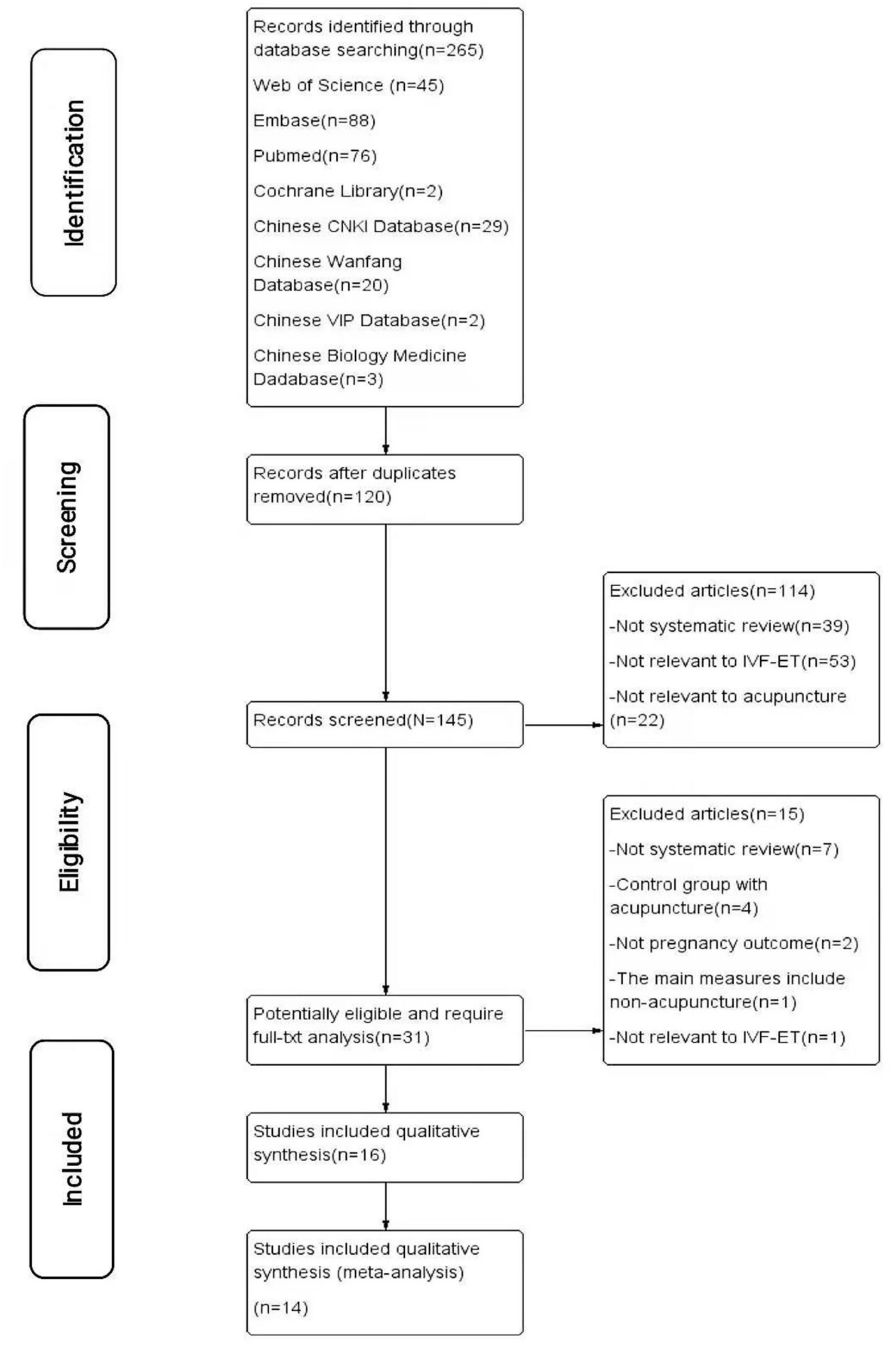
The PRISMA flow diagram of study selection.

A total of 265 records were collected from electronic databases. In total, 120 duplicates were excluded by filtration; 114 of the remaining citations were excluded by title and abstract screening, and 31 studies were assessed through the full texts. After being reviewed by two reviewers independently, 16 SRs about acupuncture for infertile women undergoing IVF-ET were included (Figure 1). The reasons for exclusion are shown in Supplementary Figure 2.

### Characteristics of Included Reviews

The sixteen included reviews (18–33) were published between 2008 and 2020. Fourteen reviews were published in English, two in Chinese. This overview included 312 original RCTs and 65,388 participants. The number of RCTs of included SRs ranged from 3 to 32 studies. As for intervention, all reviews compared acupuncture with no adjunctive treatment or sham (placebo) acupuncture. Eight reviews (20, 22, 25, 28–32) included traditional acupuncture as the main intervention. Twelve reviews (18–28, 32, 33) used electroacupuncture as an intervention measure. Eight SRs (18, 19, 21, 23, 24, 26, 27, 33) used manual acupuncture as a therapeutic intervention. Five SRs (20, 22, 26, 27, 32) included auricular acupuncture as an intervention measure. Five reviews (20–22, 25, 32) used transcutaneous electrical acupoint stimulation or laser acupuncture as the intervention. One review (30) used sham acupuncture as the control group, while others used sham acupuncture or no adjunctive treatment. Only one review (28) applied the Jadad scale for methodological quality assessment of original RCTs, and thirteen SRs (18–25, 29–33) used the Cochrane Handbook, but two reviews (26, 27) did not report the methodological tool. Table 1 shows the basic characteristics of the included SRs.

**Table 1:** Basic characteristics of the included SRs.

**Study**	**Language**	**Number of studies (total sample)**	**Nature of acupuncture**	**Nature of control interventions**	**Time of acupuncture**	**Primary outcome**	**Methodological evaluation tool**	**Data analysis methods**
Jang et al. (18)	English	3 (400)	MA/EA	Sham acupuncture or no treatment or other active control	Menstrual and ovulatory cycles	CPR	Cochrane handbook	Not applicable
Smith et al. (19)	English	20 (5,130)	MA/EA	Invasive sham control and no adjunctive treatment	Before and immediately after ET	CPR	Cochrane handbook	Meta-analysis
Gu et al. (20)	English	31 (4,450)	TA/EA/AA/TEAS	No adjunctive treatment or sham acupuncture or western medication	During COH, during the pre-embryo transfer treatment, before and immediately after ET	CPR	Cochrane handbook	Not applicable
Xie et al. (21)	English	27 (6,166)	MA/EA/AA	No adjunctive treatment or sham acupuncture	During COH, during the pre-embryo transfer treatment, before and immediately after ET	CPR/LBR	Cochrane handbook	Meta-analysis
Zhang et al. (22)	English	31 (6,098)	TA/EA/AA/TEAS	No adjunctive treatment or sham acupuncture	Not reported	CPR/LBR/BPR/OPR/MR	Cochrane handbook	Meta-analysis
Schwarze et al. (30)	English	6 (2,376)	TA	Sham acupuncture	25 or 30 min before and after ET	CPR	Cochrane handbook	Meta-analysis
Jo and Lee (23)	English	4 (430)	MA/EA	Sham acupuncture or no adjunctive treatment	Not reported	CPR/LBR	Cochrane handbook	Meta-analysis
Yang et al. (24)	Chinese	32 (4,815)	MA/EA	No adjunctive treatment or sham acupuncture	Not reported	CPR/LBR	Cochrane handbook	Meta-analysis
Qian et al. (25)	English	30 (6,344)	TA/EA/AA	No adjunctive treatment or sham acupuncture	Before and after ET, or around the time of oocyte aspiration	CPR/LBR/BPR/ OPR	Cochrane handbook	Meta-analysis
Shen et al. (26)	English	21 (5,428)	MA/LA/EA	No adjunctive treatment or sham acupuncture	The time of ET, or 25 or 30 min before and after ET	CPR	Not reported	Meta-analysis
Manheimer et al. (31)	English	16 (4,021)	TA	No adjunctive treatment or sham acupuncture	Before and after ET	CPR	Cochrane handbook	Meta-analysis
Qu et al. (32)	English	17 (3,713)	TA/AA/EA/LA	No adjunctive treatment or sham acupuncture	Before and after ET	CPR/LBR/BPR/ OPR/MR	Cochrane handbook	Meta-analysis
Zheng et al. (27)	English	24 (5,807)	MA/EA/LA	No adjunctive treatment or sham acupuncture	Before and after ET, or around the time of oocyte aspiration	CPR/LBR	Not reported	Meta-analysis
Yu (28)	Chinese	10 (2,046)	TA/AA/EA	No adjunctive treatment or sham acupuncture	25 or 30 min before and after ET	CPR/LBR/OPR	Jadad	Meta-analysis
Manheimer et al. (29)	English	7 (1,366)	TA	Sham acupuncture or no adjunctive treatment	Before and after ET	CPR/LBR/OPR	Cochrane handbook	Meta-analysis
El-Toukhy et al. (33)	English	13 (2,500)	MA/AA	No adjunctive treatment or sham acupuncture	25 or 30 min before and after ET	CPR/ LBR	Cochrane handbook	Meta-analysis

*TA, traditional acupuncture; EA, electrical acupuncture; MA, manual acupuncture; AA, auricular acupuncture; TEAS, transcutaneous electrical acupoint stimulation; LA, laser acupuncture; CPR, clinical pregnancy rate; LBR, live birth rate; BPR, biochemical pregnancy rate; OPR, ongoing pregnancy rate; MR, miscarriage rate; COH, controlled ovarian hyperstimulation*.

### Methodological Appraisal

AMSTAR-2 was used to assess the methodological quality of studies. The qualities of sixteen reviews were considered critically low, because they had more than one critical flaw (items 2, 4, 7, 9, 11, 13, and 15) with multiple non-critical weaknesses. Fourteen SRs (19–30, 32, 33) were not registered in advance, and we could not judge whether the review methods were established in advance. Only one author (19) explained their selection of the study designs for inclusion in the review. Eleven reviews (19, 22–25, 27–29, 31–33) described a comprehensive literature search strategy. Only one review (21) provided a list of excluded studies. None of the SRs described the funding sources of included RCTs. Fourteen reviews (19, 21–33) applied meta-analytical methods, and all of them explained reasons for heterogeneity reasonably. Nine studies did not declare the conflicts of interest or provide a source of funding. The details of the assessment of the quality of the included SRs are shown in Table 2.

**Table 2:** AMSTAR-2 for methodological quality of the included SRs.

**Study**	**Q1**	**Q2**	**Q3**	**Q4**	**Q5**	**Q6**	**Q7**	**Q8**	**Q9**	**Q10**	**Q11**	**Q12**	**Q13**	**Q14**	**Q15**	**Q16**	**Ranking of quality**
Jang et al. (18)	Y	Y	N	PY	Y	Y	N	Y	Y	N	N/A	N/A	Y	Y	N/A	Y	Critically low
Smith et al. (19)	Y	N	Y	Y	Y	Y	N	PY	Y	N	Y	Y	Y	Y	Y	N	Critically low
Gu et al. (20)	Y	N	N	PY	Y	Y	N	Y	Y	N	N/A	N/A	Y	Y	N/A	N	Critically low
Xie et al. (21)	Y	N	N	Y	Y	Y	Y	Y	Y	N	Y	Y	Y	Y	Y	Y	Critically low
Zhang et al. (22)	Y	N	N	Y	Y	Y	N	Y	Y	N	Y	Y	Y	Y	N	Y	Critically low
Schwarze et al. (30)	Y	N	N	PY	Y	Y	N	PY	Y	N	Y	Y	Y	Y	Y	N	Critically low
Jo and Lee (23)	Y	N	N	Y	Y	Y	N	PY	Y	N	Y	Y	Y	Y	N	N	Critically low
Yang et al. (24)	Y	N	N	Y	Y	Y	N	Y	Y	N	Y	Y	Y	Y	Y	Y	Critically low
Qian et al. (25)	Y	N	N	Y	Y	Y	N	Y	Y	N	N	Y	Y	Y	N	Y	Critically low
Shen et al. (26)	Y	N	N	PY	Y	Y	N	PY	N	N	Y	N	Y	Y	N	N	Critically low
Manheimer et al. (31)	Y	Y	N	Y	Y	Y	N	PY	Y	N	Y	Y	Y	Y	Y	Y	Critically low
Qu et al. (32)	Y	N	N	Y	Y	Y	N	Y	Y	N	Y	Y	Y	Y	N	N	Critically low
Zheng et al. (27)	Y	N	N	Y	Y	Y	N	PY	N	N	Y	N	Y	Y	Y	N	Critically low
Yu (28)	Y	N	N	Y	Y	Y	N	PY	Y	N	Y	N	Y	Y	Y	N	Critically low
Manheimer et al. (29)	Y	N	N	Y	Y	Y	N	PY	Y	N	Y	Y	Y	Y	Y	Y	Critically low
El-Toukhy et al. (33)	Y	N	N	Y	Y	Y	N	PY	PY	N	Y	Y	Y	Y	Y	N	Critically low

### Risk of Bias of Included Systematic Reviews

**Figure 2:**
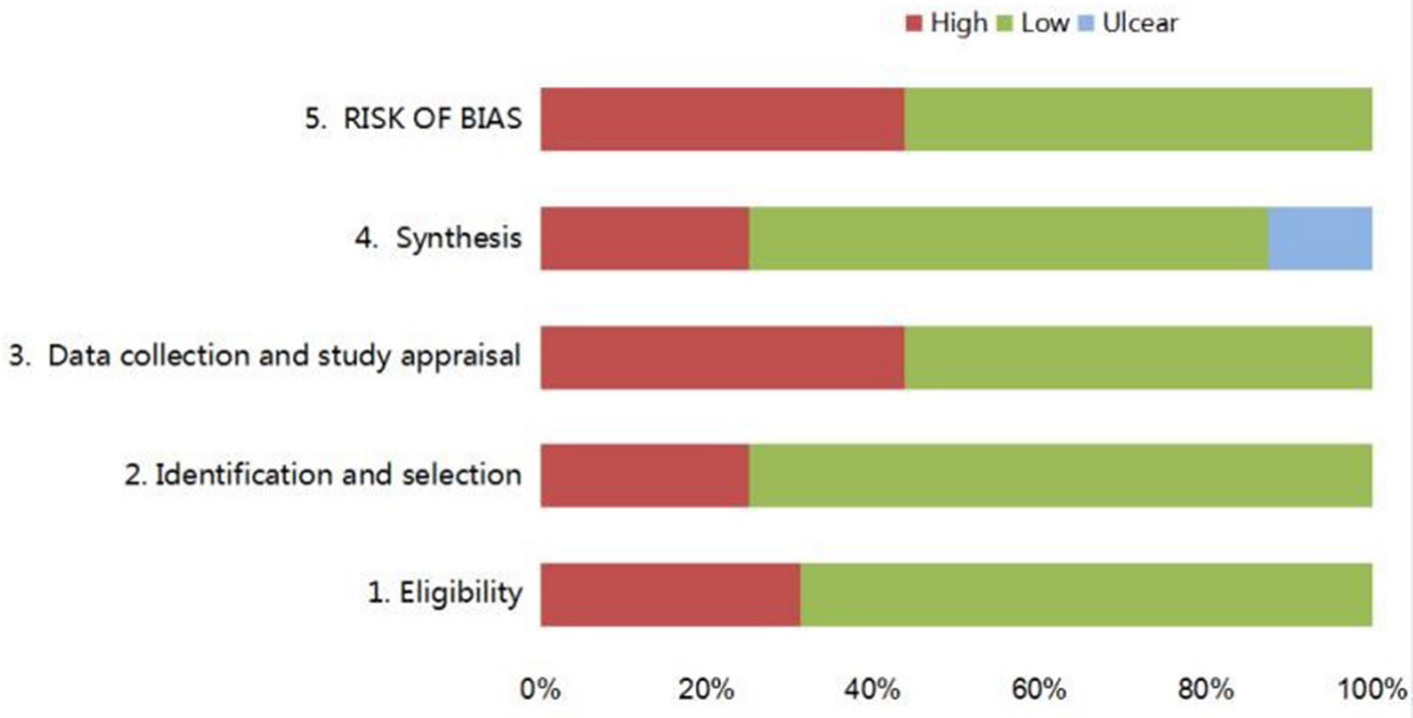
Graphical presentation of risk of bias of the included SRs.

The assessment of the risk of bias of each review is shown in Table 3 and Figure 2. The final phase considered the overall risk of bias of SRs, and nine SRs (56.25%) were rated with a low-risk of bias. Seven SRs (43.75%) were rated with a high-risk of bias, and the main reason for the risk of bias in SRs was the failure to adequately explain and deal with the risk of bias in their results.

**Table 3:** Suggested tabular presentation for ROBIS results.

**Review**	**Phase 2**				**Phase 3**
	**1. Study eligibility criteria**	**2. Identification and selection of studies**	**3. Data collection and study appraisal**	**4. Synthesis and findings**	**Risk of bias in the review**
Jang et al. (18)	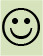	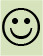	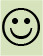	?	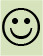
Smith et al. (19)	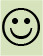	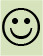	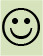	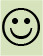	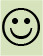
Gu et al. (20)	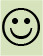	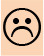	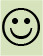	?	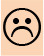
Xie et al. (21)	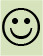	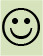	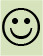	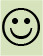	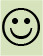
Zhang et al. (22)	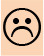	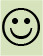	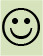	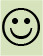	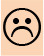
Schwarze et al. (30)	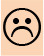	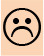	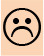	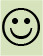	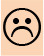
Jo and Lee (23)	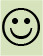	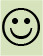	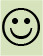	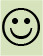	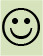
Yang et al. (24)	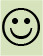	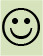	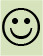	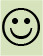	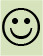
Qian et al. (25)	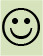	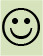	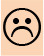	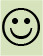	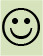
Shen et al. (26)	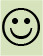	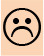	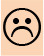	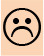	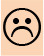
Manheimer et al. (31)	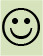	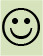	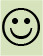	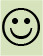	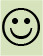
Qu et al. (32)	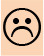	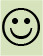	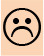	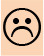	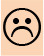
Zheng et al. (27)	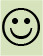	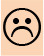	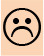	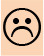	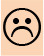
Yu (28)	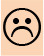	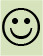	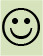	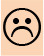	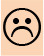
Manheimer et al. (29)	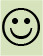	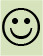	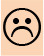	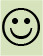	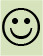
El-Toukhy et al. (33)	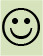	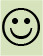	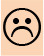	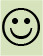	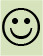

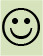
, *low risk;* 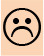, *high risk; ?, unclear risk*.

### GRADE for Quality of Evidence Profile

Fourteen reviews (19, 21–33) included 35 outcomes that were related to the effectiveness of acupuncture for IVF-ET. The risk of bias, imprecision, and reporting bias were the main reasons for downgrading. There was high or moderate or low or very low-grade evidence to indicate that acupuncture might improve the CPR and LBR when acupuncture was performed on the day of ET. The qualities of the evidence are shown in Table 4.

**Table 4:** GRADE for quality of evidence profile.

**Outcomes**	**Study**	**Number of studies**	**Effect (95% CI)**	**Risk of bias**	**Inconsistency**	**Indirectness**	**Imprecision**	**Other considerations**	**Quality of evidence**
CPR	Smith et al. (19)	20	RR 1.32 (1.07, 1.62)	Not serious	Not serious	Not serious	Not serious	None	High
	Xie et al. (21)	27	RR 1.21 (1.07, 1.38)	Serious	Not serious	Not serious	Not serious	None	Moderate
	Zhang et al. (22)	31	RR 1. 19 (1.06, 1.34)	Serious	Not serious	Not serious	Not serious	None	Moderate
	Schwarze et al. (30)	6	RR 0. 87 (0.77, 0.98)	Not serious	Not serious	Not serious	Not serious	None	High
	Jo and Lee (23)	4	RR 1.35 (1.05, 1.74)	Serious	Not serious	Not serious	Serious	None	Low
	Yang et al. (24)	14	RR 1.43 (1.15, 1.77)	Serious	Not serious	Not serious	Not serious	Reporting bias	Low
	Qian et al. (25)	30	OR 1.26 (1.06, 1.50)	Serious	Not serious	Not serious	Serious	Reporting bias	Very low
	Shen et al. (26)	10	RR 1.24 (1.02, 1.50)	Serious	Not serious	Not serious	Not serious	Reporting bias	Low
	Manheimer et al. (31)	7	OR 1.65 (1.27, 2.14)	Serious	Not serious	Not serious	Not serious	None	Moderate
	Qu et al. (32)	17	RR 1. 09 (0.94, 1.26)	Serious	Not serious	Not serious	Serious	Reporting bias	Very low
	Zheng et al. (27)	23	OR 1.22 (1.01, 1.47)	Serious	Not serious	Not serious	Not serious	None	Moderate
	Yu (28)	11	RR 1.34 (1.09, 1.66)	Serious	Not serious	Not serious	Not serious	Reporting bias	Low
	Manheimer et al. (29)	7	OR 1.65 (1.27, 2.14)	Not serious	Not serious	Not serious	Not serious	Reporting bias	Moderate
	El-Toukhy et al. (33)	8	RR 1.23 (0.96, 1.58)	Not serious	Not serious	Not serious	Serious	None	Moderate
LBR	Zhang et al. (38)	12	RR 1.36 (1.09, 1.69)	Not serious	Not serious	Not serious	Not serious	None	High
	Jo and Lee (23)	1	RR 1.61 (0.73, 3.58)	Not serious	Not serious	Not serious	Serious	Reporting bias	Low
	Yang et al. (24)	8	RR 1.18 (0.89, 1.58)	Not serious	Not serious	Not serious	Serious	Reporting bias	Low
	Qian et al. (25)	9	OR 1.17 (0.80, 1.72)	Serious	Not serious	Not serious	Serious	Reporting bias	Very low
	Qu et al. (32)	6	RR 1.42 (0.92, 2.20)	Serious	Not serious	Not serious	Serious	Reporting bias	Very low
	Zheng et al. (27)	6	OR 1.09 (0.74, 1.60)	Serious	Not serious	Not serious	Serious	Reporting bias	Very low
	Manheimer et al. (29)	4	OR 1. 91 (1.39, 2.64)	Not serious	Not serious	Not serious	Not serious	Reporting bias	Moderate
	El-Toukhy et al. (33)	5	RR 1.34 (0.85, 2.11)	Not serious	Not serious	Not serious	Serious	None	Moderate
	Yu (28)	6	RR 1.28 (0.91, 1.79)	Serious	Not serious	Not serious	Serious	Reporting bias	Very low
BPR	Zhang et al. (38)	12	RR 1.12 (0.92, 1.35)	Serious	Not serious	Not serious	Serious	None	Low
	Qian et al. (25)	17	OR 1.06 (0.82, 1.37)	Serious	Not serious	Not serious	Serious	Reporting bias	Very low
	Qu et al. (32)	9	RR 1.01 (0.84, 1.20)	Not serious	Not serious	Not serious	Serious	Reporting bias	Low
OPR	Zhang et al. (22)	9	RR 1.21 (0.95, 1.55)	Serious	Not serious	Not serious	Serious	None	Low
	Qian et al. (25)	10	OR 1.14 (0.87, 1.48)	Serious	Not serious	Not serious	Serious	Reporting bias	Very low
	Qu et al. (32)	8	RR 1.20 (0.93, 1.56)	Serious	Not serious	Not serious	Serious	Reporting bias	Very low
	Manheimer et al. (29)	5	OR 1.87 (1.40, 2.49)	Not serious	Not serious	Not serious	Not serious	Reporting bias	Moderate
	Yu (28)	6	RR 1.28 (0.91, 1.79)	Serious	Not serious	Not serious	Serious	Reporting bias	Very low
MR	Zhang et al. (22)	12	RR 0.89 (0.67, 1.20)	Serious	Not serious	Not serious	Serious	None	Low
	Qu et al. (32)	5	RR 0.94 (0.67, 1.33)	Serious	Not serious	Not serious	Serious	Reporting bias	Very low

### Effect of the Interventions

#### Acupuncture vs. Sham Acupuncture

**Figure 3:**
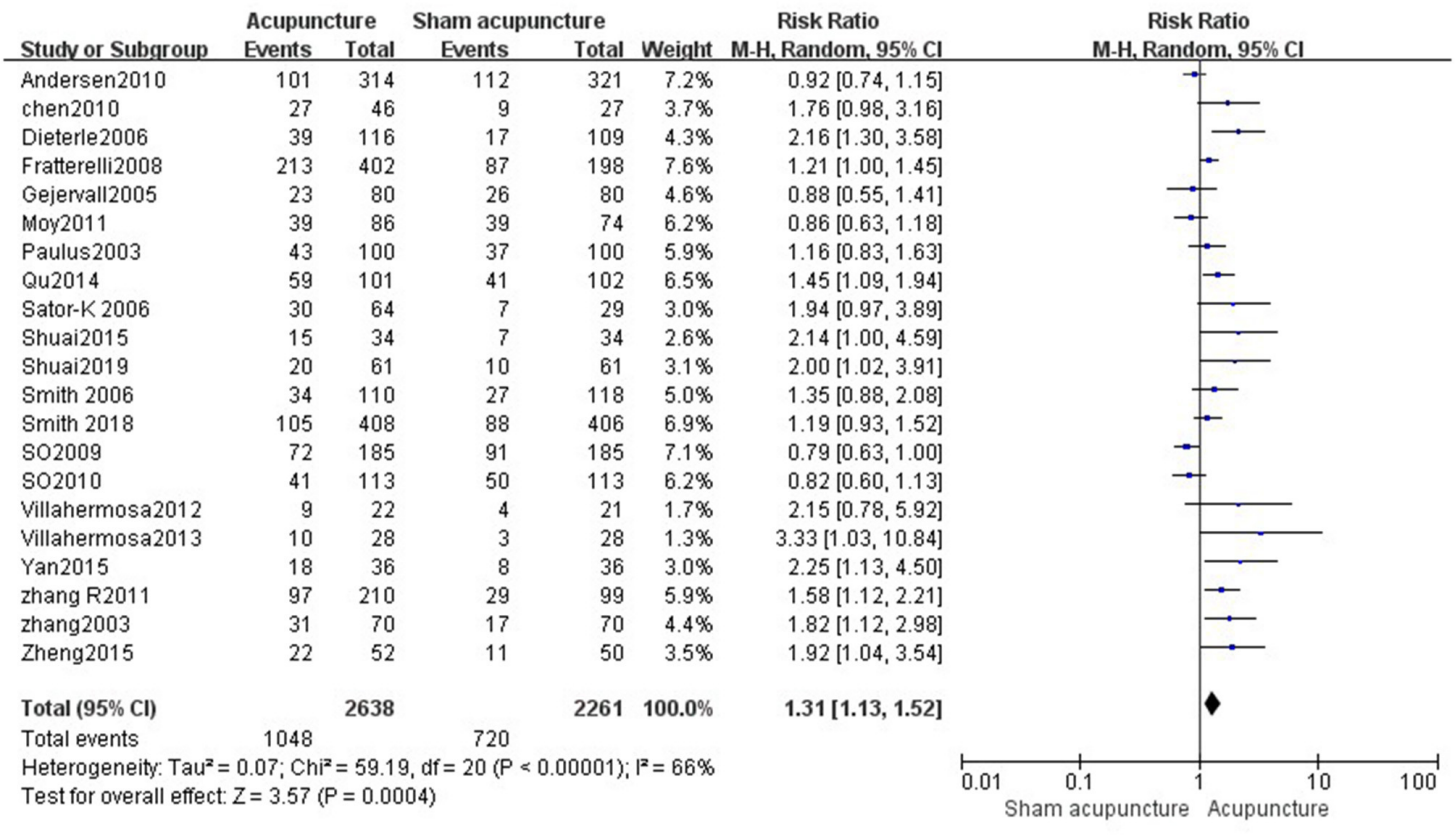
Acupuncture vs. sham acupuncture on the clinical pregnancy rate.

Fourteen reviews (19, 21–33) encompassing 21 primary RCTs (4,899 participants) suggested that acupuncture was superior to sham acupuncture in increasing the CPR of IVF-ET (RR = 1.31, 95% CI: 1.13–1.52, *p* = 0.0004). However, there was substantial heterogeneity for the CPR (*I*^2^ = 66%) (Figure 3). There was no statistical difference between the acupuncture and sham acupuncture groups for improving LBR (RR = 0.92, 95% CI: 0.83–1.00, *p* = 0.06, 10 RCTs, 3,171 participants) or OPR (RR = 1.13, 95% CI: 0.90–1.41, *p* = 0.30, 8 RCTs, 2,793 participants) (**Figures 5**, **6**). The benefits for reducing MR (OR = 1.20, 95% CI: 0.87–1.65, *p* = 0.28, 7 RCTs, 2,698 participants) or BPR (RR = 1.08, 95% CI: 0.87–1.32, *p* = 0.49, 8 RCTs, 2,581participants) were not significant between the acupuncture and control groups (**Figures 7**, **8**).

#### Acupuncture vs. No Adjunctive Treatment

**Figure 4:**
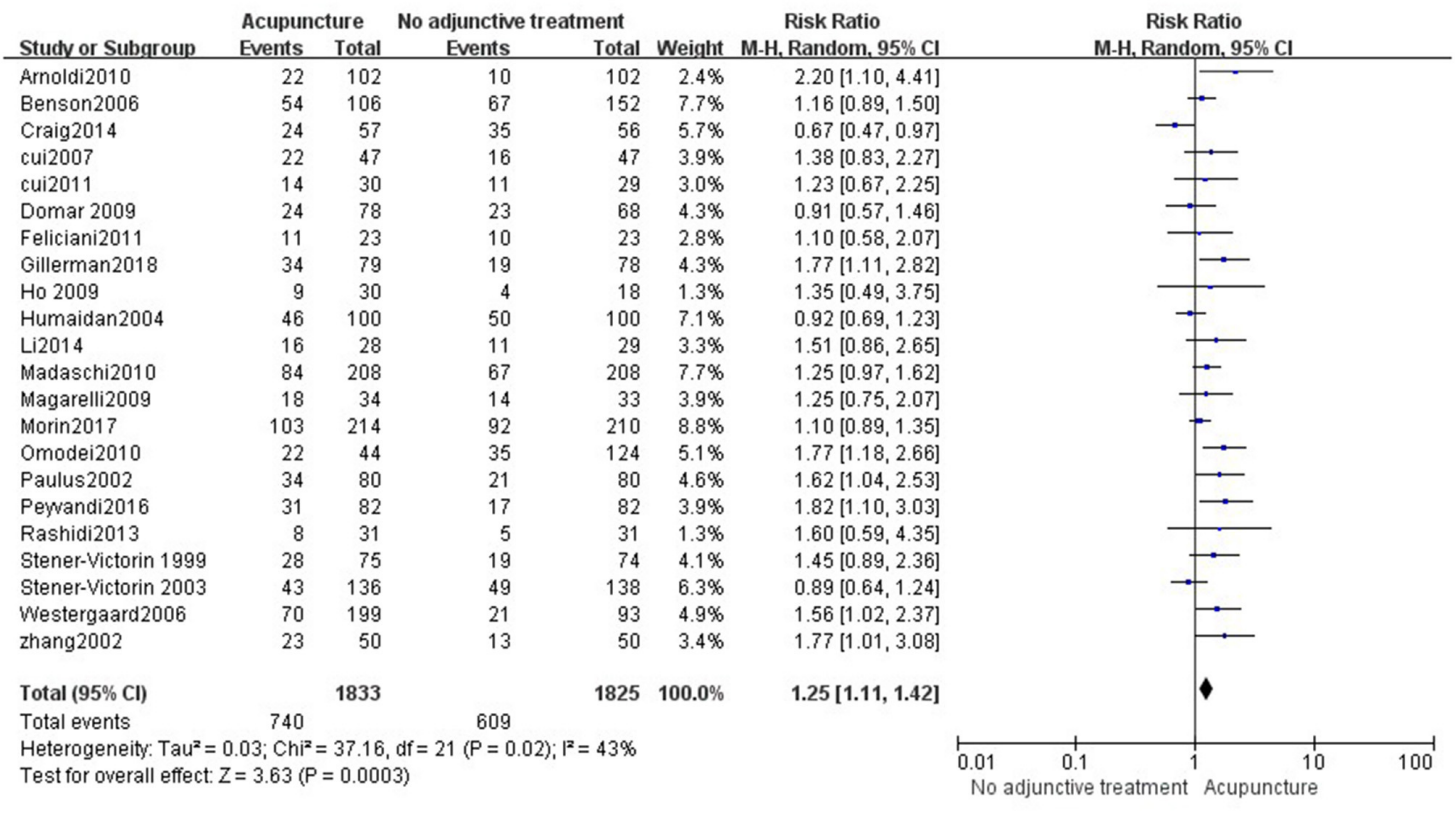
Acupuncture vs. no adjunctive treatment on the clinical pregnancy rate.

**Figure 5:**
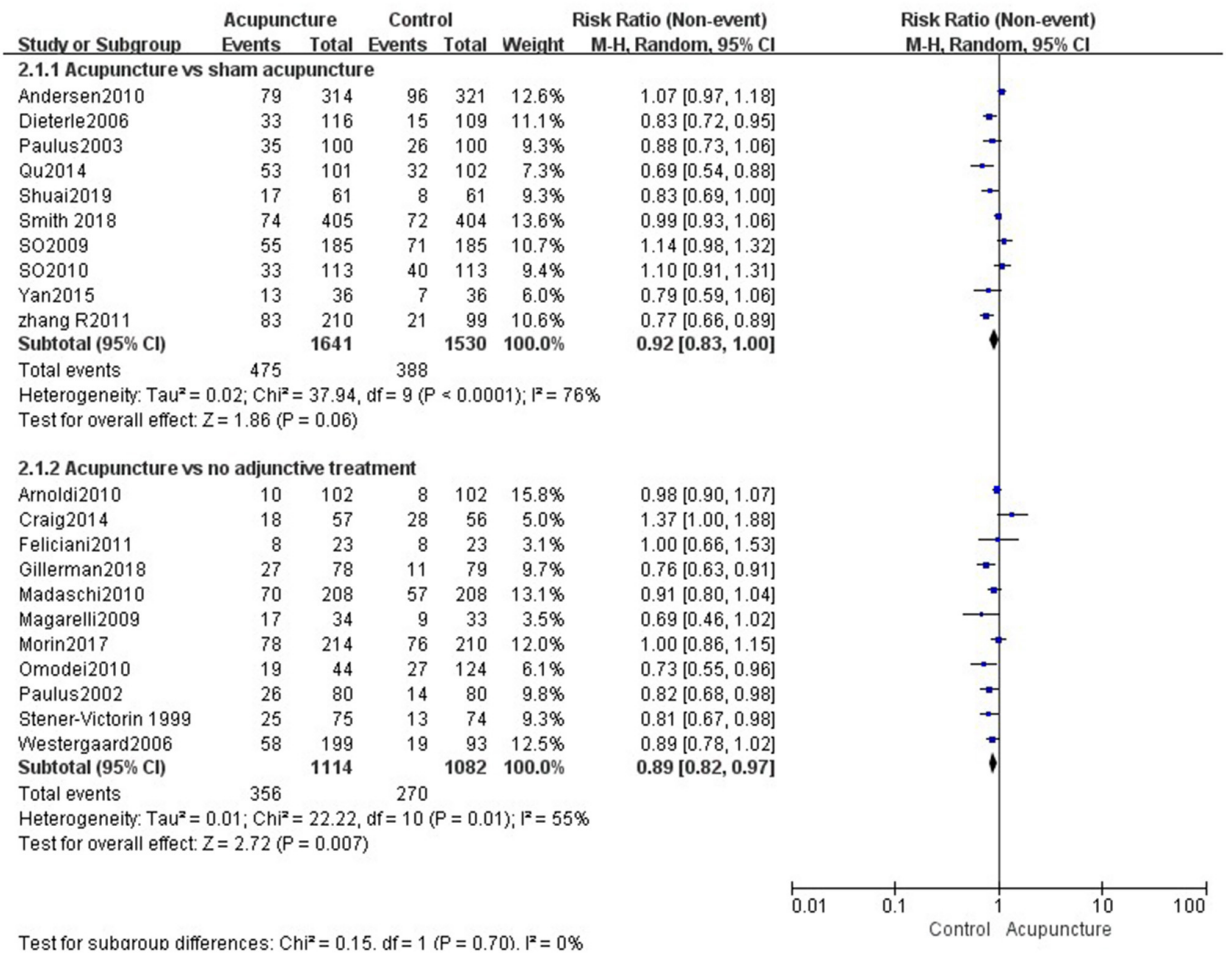
Effects of acupuncture on the live birth rate.

**Figure 6:**
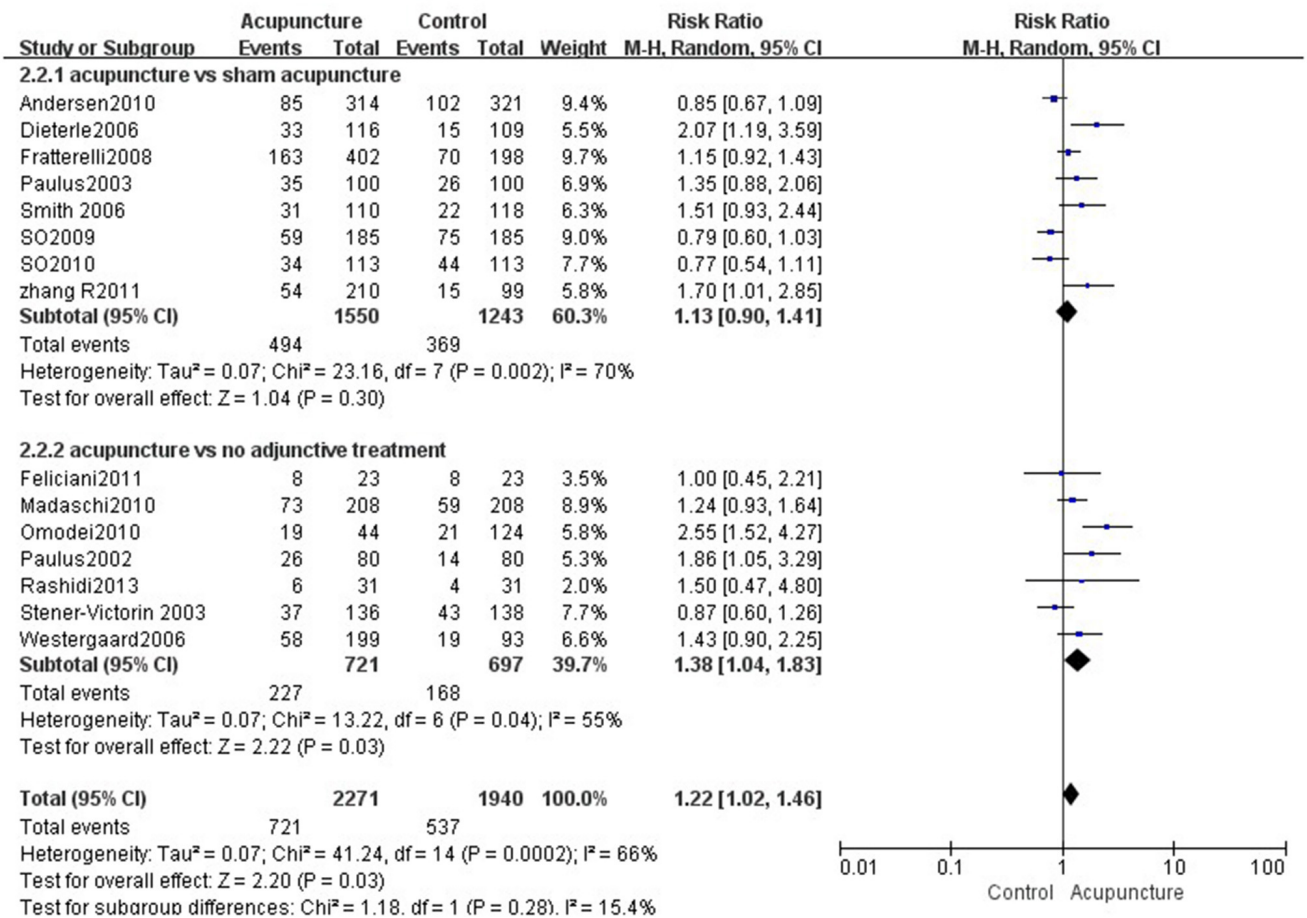
Effects of acupuncture on the ongoing pregnancy rate.

**Figure 7:**
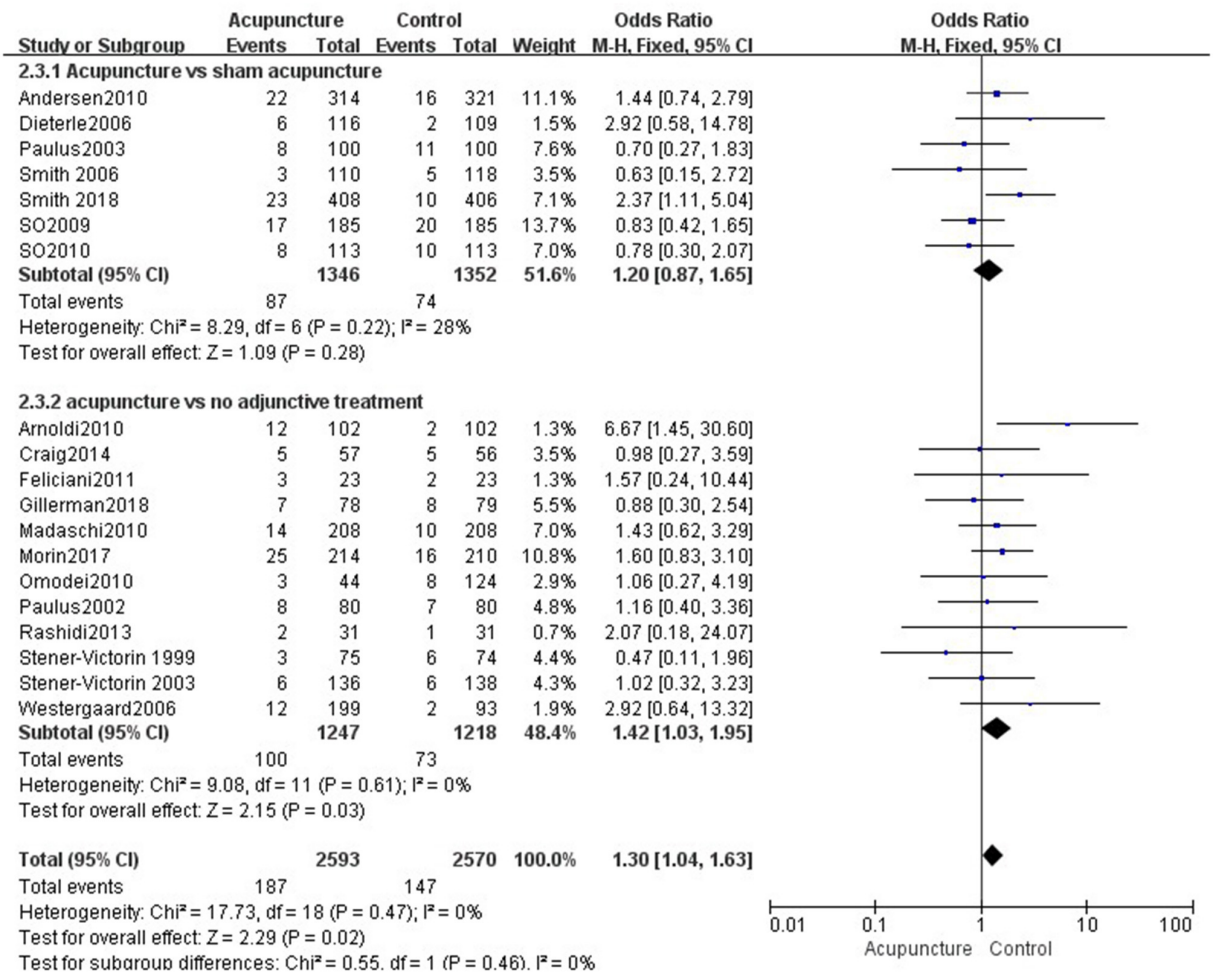
Effects of acupuncture on the miscarriage rate.

**Figure 8:**
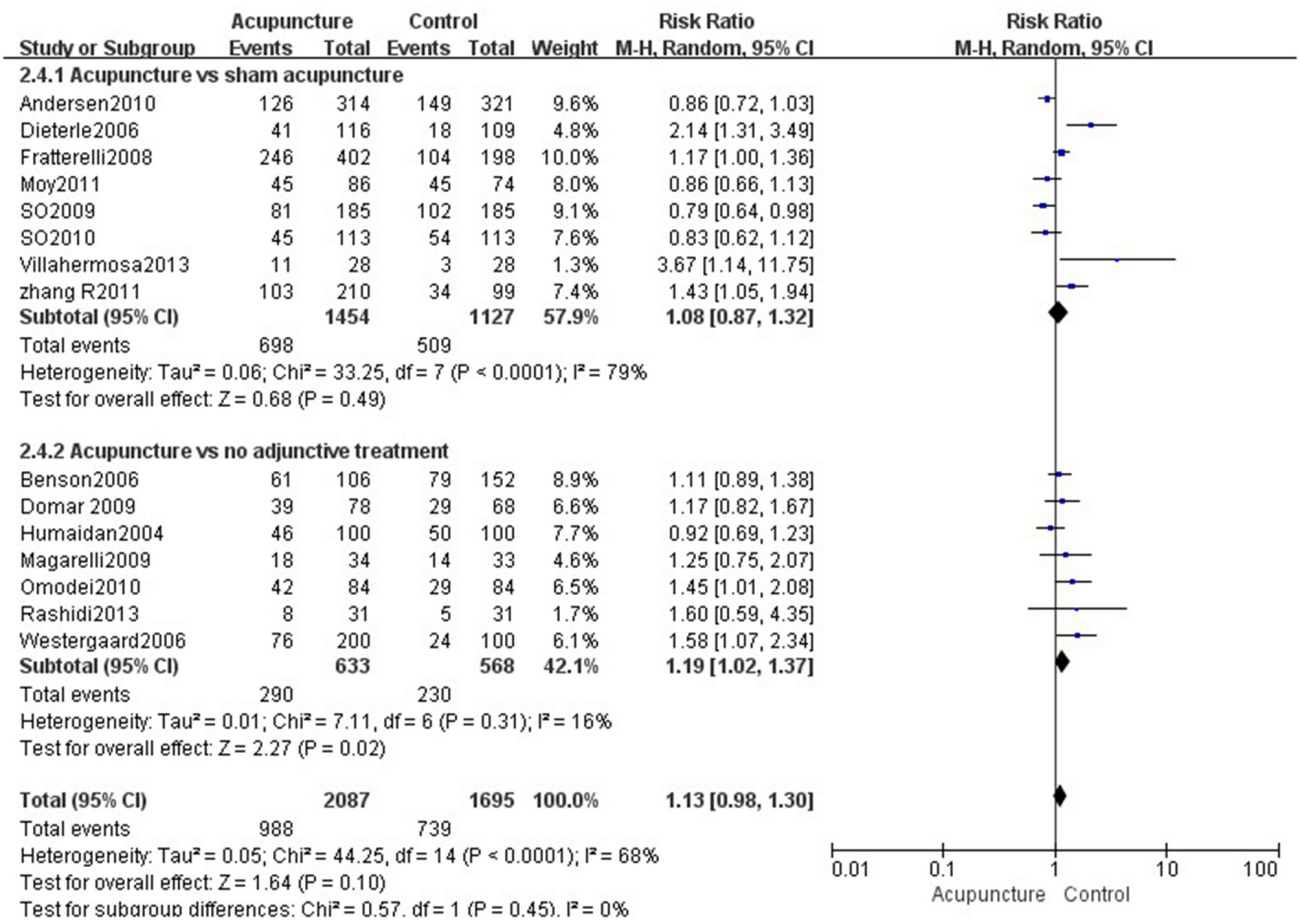
Effects of acupuncture on the biochemical pregnancy rate.

Data were obtained from 22 RCTs (3,658 participants) out of the 14 included reviews. When using the random-effects model, the pooled results showed that acupuncture groups were significantly better than no adjunctive treatment group in improving the CPR (RR = 1.25, 95% CI: 1.11–1.42, *p* = 0.0003; Figure 4). The results of 11 RCTs (2,196 participants) suggested that acupuncture was not better than no adjunctive treatment in the LBR (RR = 0.89, 95% CI: 0.82–0.97, *p* = 0.007; Figure 5). OPR data from 7 RCTs out of the 14 included reviews were available (1,418 participants). A significant difference in the OPR was observed when the random-effects model was used (RR = 1.38, 95% CI: 1.04–1.83, *p* = 0.03; Figure 6). Moreover, acupuncture was more superior than no adjunctive treatment on MR reduction (OR = 1.42, 95% CI: 1.03–1.95, *p* = 0.03, 12 RCTs, 2,465 participants) (Figure 7). The pooled results showed a significant difference in reducing the BPR (RR = 1.19, 95% CI: 1.02–1.37, *p* = 0.02) between acupuncture groups and no adjunctive treatment groups (Figure 8).

#### Subgroup Analysis on Clinical Pregnancy Rate

Sensitivity analysis was conducted by examining individual studies, the pooled results were not affected. Age below 35 years was a significant factor in the CPR (RR = 1.41, 95% CI: 1.15–1.74). Studies that used the invasive sham control showed a significant difference in the CPR (RR = 1.77, 95% CI: 1.09–2.86), and non-invasive sham control also significantly improved the CPR (RR = 1.28, 95% CI: 1.06–1.53). The duration of infertility over 4 years was not conducive to improving the CPR (RR = 0.81, 95% CI: 0.71–0.93). The number of transplanted embryos more than two (RR = 0.79, 95% CI: 0.70–0.88) and the absences of sham acupoint controls (RR = 0.82, 95% CI: 0.74–0.92) were detrimental to the CPR. Age over 35 years, duration of infertility <4 years, and sham points were not significant modifiers of the CPR. Table 5 shows the results of the subgroup analysis of outcomes regarding the CPR.

**Table 5:** Subgroup analysis on clinical pregnancy rate.

**Characteristic**	**Subgroup analyses**				**Heterogeneity**	
	**No. of subjects**	**No. of studies**	**RR (95% CI)**	***P*-value**	***I^**2**^***	***P***
**Age**						
≥35 years	1,796	6	1.15 (0.85, 1.54)	0.36	72%	0.003
<35 years	2,430	13	1.41 (1.15, 1.74)	0.001	65%	0.0007
**Duration of infertility**						
≥4 years	1,627	11	0.81 (0.71, 0.93)	0.003	69%	0.0004
<4 years	863	2	0.98 (0.84, 1.13)	0.76	58%	0.13
**No. of embryos transferred**						
≥2	1,093	6	0.79 (0.70, 0.88)	<0.0001	33%	0.19
<2	2,307	6	1.02 (0.92, 1.13)	0.07	61%	0.02
**Type of sham**						
Invasive	629	6	1.77 (1.09, 2.86)	0.02	72%	0.003
Non-invasive	3,510	13	1.28 (1.06, 1.53)	0.009	69%	0.0001
**Sham points**						
Yes	1,245	4	0.94 (0.86, 1.03)	0.16	18%	0.30
No	2,894	15	0.82 (0.74, 0.92)	0.0004	71%	<0.0001

### Adverse Effects

A total of five reviews (18, 19, 22, 29, 31) mentioned adverse events. Only three SRs (18, 19, 22) reported adverse events, including mild allergy, nausea, drowsiness, headache, chest pain, dizziness, and fatigue, while the other two SRs (29, 31) reported no serious adverse events.

## Discussion

### Summary of Main Findings

This updated overview of 16 SRs summarized the clinical evidence on the effectiveness and safety of acupuncture for infertile women undergoing IVF-ET from 312 primary studies that included 65,388 participants, and evaluated the methodological quality and quality of evidence. The current evidence indicated that acupuncture appeared to be superior to the control group in improving the CPR of IVF-ET. However, the methodological qualities of SRs were critically low, and the GRADE for the quality of evidence profile was suboptimal. By using the ROBIS tool, seven SRs were rated with a high-risk bias. Some SRs showed that acupuncture might be increasing LBR and OPR, but the sample was insufficient. No significant difference was found in reducing BPR and MR between groups. High-quality RCTs with large sample sizes were necessary to demonstrate the clinical effectiveness of acupuncture for IVF-ET.

In this overview, we used AMSTAR-2 to assess the methodological quality of SRs. The confidence of the overview was critically low. In some reviews using AMSTAR-2, we also found that the overall confidence in the results was rated as critically low (38–40). AMSTAR-2 was updated in 2017, so previous SRs might not have covered some items of AMSTAR-2. It might be one reason why most of the SRs were critically low as assessed by AMSTAR-2. The methodological quality of SRs was limited by the lack of data on registration and funding, comprehensive search strategy, a list and justification of excluded articles, and explanation of the risk of bias. Future research should pay attention to the above issues. The GRADE system was used to assess the evidence quality of the included SRs. The strength of evidence was moderate or low or very low for most outcomes. Most of the outcome indicators were demoted because of the bias in random, distributive hiding, or blind studies. Due to the characteristics of acupuncture, it was difficult for patients to achieve blinding, so it was important to separate researchers.

Our re-meta-analysis finds that acupuncture in IVF-ET trials appears to increase CPR, OPR, and LBR compared with no adjunctive treatment. The differences are not significant compared with sham acupuncture in increasing OPR and LBR. Since the quality of the methodology of all the SRs included is critically low, there is a high probability that the result of the re-meta-analysis is biased. There is significant heterogeneity in these clinical studies of acupuncture. Almost every trial has a different approach design in terms of interventions, the timing of treatments, controls, and outcome measures. Subgroup analysis is used to explore the factors that may affect the CPR of acupuncture. Age below 35 years is a significant factor on reproductive outcomes. Non-invasive sham control also shows a significant difference, so it is also important to estimate the placebo effect of sham acupuncture in acupuncture IVF-ET trials. Currently, most trials adopt the protocol of Paulus (41), involving acupuncture treatments before and after ET. However, the quality of the embryo is essential, but acupuncture only before and after ET has significant limitations.

### Strengths and Limitations

To reduce the risk of bias, we only included the SRs of randomized trials. We evaluated the methodological quality of the included SRs by using the AMSTAR-2 tool, which was the latest available assessment tool. We assessed the GRADE score to determine the strength of evidence. We excluded the overlapping RCTs and performed a re-meta-analysis of the primary RCTs. To minimize potential bias in the overview process, we used more than two reviewers in the literature screening, data extraction, and quality assessment.

The latest RCTs were unlikely to be included in the recently published SRs, so there was some publication bias. Almost every IVF-ET trial had a different methodological design, which tended to cause severe heterogeneity, which limited the ability to interpret aggregate estimates. We collected evidence for acupuncture in IVF-ET, but we could not separate the different types of acupuncture intervention and timing of treatments.

### Opportunities for Future Research

There is some debate about the effectiveness of acupuncture to treat infertile women undergoing IVF-ET. Acupuncture appears to be superior to the control group in improving the CPR of IVF-ET, but the evidence should be treated cautiously because of the methodological flaws. Recommendations for further studies are as follows: Most RCTs are considered to have an unclear risk of bias in the domains of allocation concealment and selective outcome reporting due to poor reporting, so RCTs should comply with the relevant guidelines. In most of the included studies, acupuncture was performed within a few days or hours before and after ET. So we can perform acupuncture during COH to observe the effect of acupuncture on the number of eggs obtained and oocyte quality. The duration of acupuncture may also influence the efficacy of acupuncture. Increasing the duration of insertion may increase the cumulative effect of acupuncture. Some reviews found that women with a history of multiple IVF-ET failures would benefit more from the effect of acupuncture, so more relevant RCTs should be conducted to estimate the effectiveness of acupuncture in the future. Acupuncture and placebo acupuncture touching the skin would evoke activity in cutaneous afferent nerves and leading to the “limbic touch response,” so they were equally effective (42). Therefore, it is also necessary to estimate the clinical effects of sham acupuncture in acupuncture IVF-ET trials. The difference of non-specific effect between acupuncture and sham acupuncture can be reduced to the maximum extent through the effective blind and random method. An advance registration contributes to improving transparency and minimizes potential bias. Only two SRs reported the protocol or registration number, so advance registration should be encouraged.

## Conclusion

This study reviews the SRs of acupuncture for infertile women undergoing IVF-ET. Based on the current evidence, acupuncture appears to be beneficial to increase the CPR in women undergoing IVF-ET. However, there are severe heterogeneity and methodological quality defects, which limit the reliability of results. Further, high-quality primary studies are still needed.

## Data Availability Statement

The original contributions presented in the study are included in the article/Supplementary Material, further inquiries can be directed to the corresponding author/s.

## Author Contributions

XW and YW participating in study design, critical dialogue, data analysis, and article writing. SW contributed to the design of this study, participated in critical dialogue, and revised the manuscript. BH, YC, and NZ contributed to the literature search and extracted data. ML proofread the manuscript. All authors contributed to the article and approved the submitted version.

## Conflict of Interest

The authors declare that the research was conducted in the absence of any commercial or financial relationships that could be construed as a potential conflict of interest.

## Appendix

The following search strategy was used for EMBASE and was modified to suit other databases.

#1. “*in vitro* fertilization”/exp OR “*in vitro* fertilization” (94,173)

#2. “intracytoplasmic sperm injection” (22,292)

#3. “embryo transfer” (34,983)

#4. “assisted reproductive techniques” (2,769)

#5. #1 OR #2 OR #3 OR #4 (9,7692)

#6. acupuncture (51,982)

#7. “acupuncture therapy” (1,780)

#8. “acupuncture points” (2,067)

#9. “electroacupuncture” (8,057)

#10. “acupressure” (2,405)

#11. “acupuncture analgesia” (2,164)

#12. #6 OR #7 OR #8 OR #9 OR #10 OR #11 (54,401)

#13. “review it” (2,912)

#14. “systematic review” (330,910)

#15. “meta analysis” (290,000)

#16. “systematic* review*” (357,380)

#17. “meta analy*” (307,640)

#18. #13 OR #14 OR #15 OR #16 OR #17 (504,371)

#19. #5 AND #12 AND #18 (88).
