# Supplementary material for: An Overview of Systematic Reviews of Acupuncture for Infertile Women Undergoing in vitro Fertilization and Embryo Transfer
Source: Front Public Health. 2021 Apr 20;9:651811. doi: 10.3389/fpubh.2021.651811 (PMC8096176; doi:10.3389/fpubh.2021.651811)
Supplement: Supplementary file 1 [file Data_Sheet_1.docx]

Supplementary Material.

**Supplementary Figure 1**

**
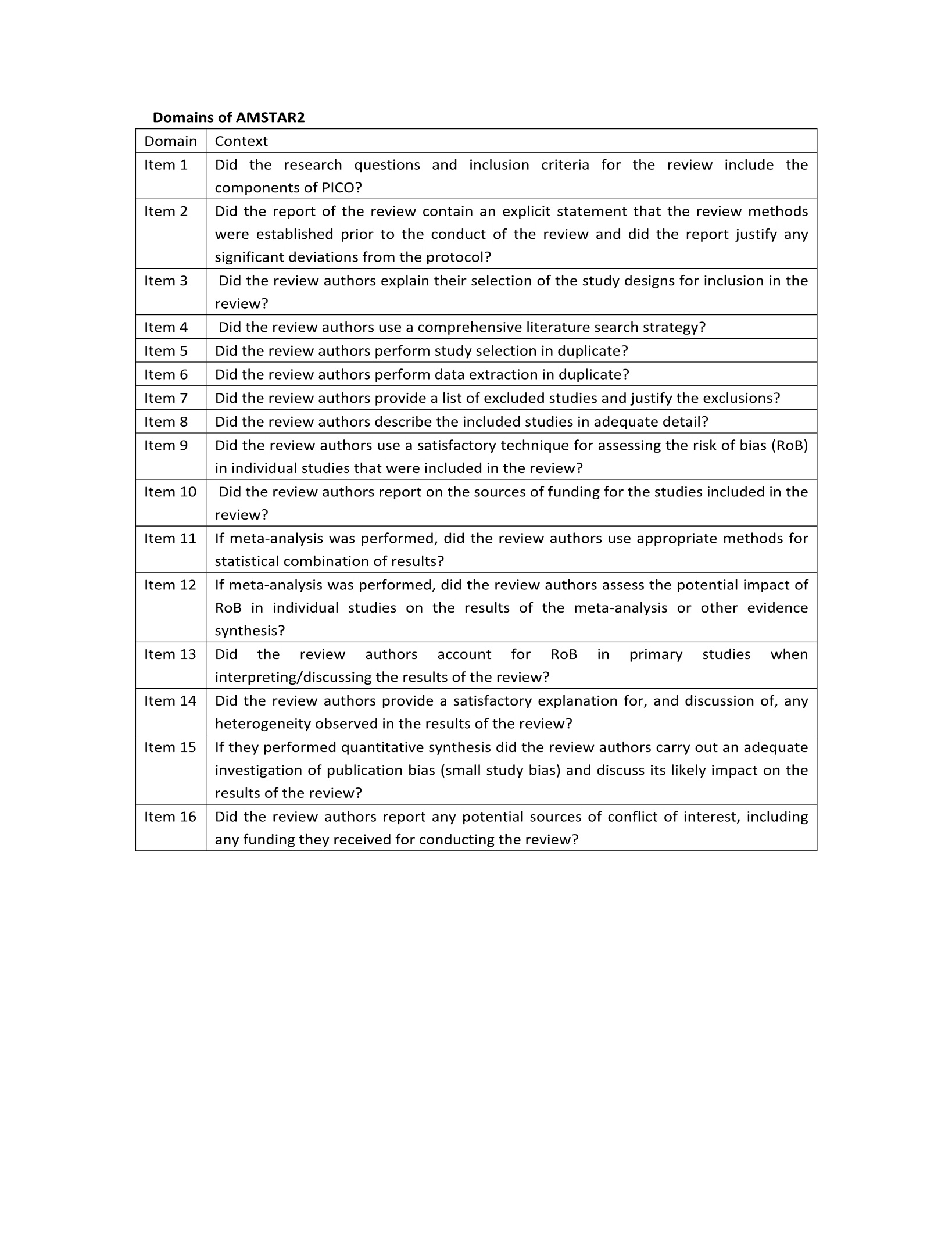
**

**Supplementary Figure 2.**


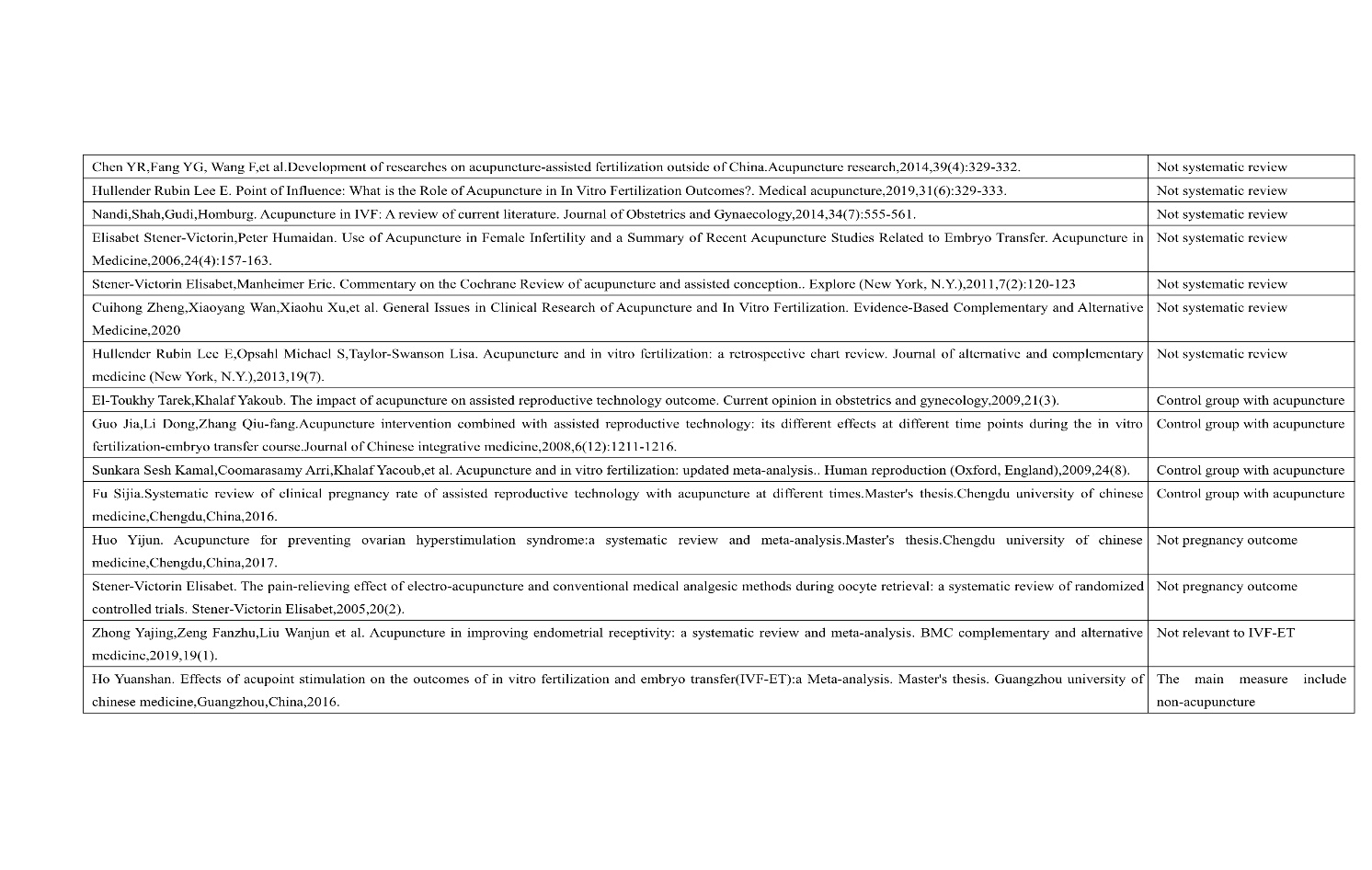


**Supplementary table 1 characteristics of acupuncture and sham acupuncture interventions**

| **Study** | **Age** | **Duration of infertility** | **No. of embryos transferred** | **Type of sham** | **Sham points** |
| --- | --- | --- | --- | --- | --- |
| Andersen2010 | 31 | 2.5 | 1.39 | N | N |
| Chen2010 | - | - | - | Y | N |
| Dieterle2006 | 34.9 | 5.4 | 2.6 | Y | N |
| Fratterelli2008 | - | - | - | - | - |
| Gejervall2005 | 33.9 | - | 1.56 | - | - |
| Moy2011 | 33.2 | - | 2.15 | Y | Y |
| Paulus2003 | 32.6 | - | - | N | N |
| Sator-K 2006 | 33.8 | - | - | N | N |
| Smith 2006 | 36 | 2.9 | - | N | Y |
| Smith 2018 | 35.4 | - | 1.32 | N | Y |
| SO2009 | 36 | 4 | 1.89 | N | N |
| SO2010 | 36 | 5 | 1.93 | N | N |
| Villahermosa2012 | 34.2 | 4 | - | Y | Y |
| Villahermosa2013 | 36.2 | 4.5 | 2.13 | Y | N |
| Yan2015 | 29.85 | 4.91 | - | Y | N |
| Zhang R2011 | 32.6 | - | 2.5 | N | N |
| Zhang2003 | 31.8 | 4.8 | 2.1 | N | N |
| Zheng2015 | 36.08 | 4.44 | 1.94 | N | N |
| Qu2014 | 30.95 | 4.97 | 2.08 | N | N |
| Shuai2019 | 31.23 | 5.09 | - | N | N |
| Shuai2015 | 29.47 | 4.56 | - | N | N |
